# Current Status of Out-of-Hospital Management of Cancer Patients and Awareness of Internet Medical Treatment: A Questionnaire Survey

**DOI:** 10.3389/fpubh.2021.756271

**Published:** 2021-12-14

**Authors:** Shuang Dai, Xiaoqin Liu, Xi Chen, Jun Bie, Chi Du, Jidong Miao, Ming Jiang

**Affiliations:** ^1^Department of Lung Cancer Center, West China Hospital Sichuan University, Chengdu, China; ^2^Department of Oncology, First People's Hospital of Jintang County, Chengdu, China; ^3^Department of Cancer Center, Yibin Second People's Hospital, Yibin, China; ^4^Department of Oncology, Nanchong Central Hospital, Nanchong, China; ^5^Department of Oncology, Hospital of Zhi Zhong Zhi Zhou & Cancer Hospital of Neijiang, Neijiang, China; ^6^Department of Oncology, Zigong Fourth People's Hospital, Zi Gong, China; ^7^Department of Head and Neck Oncology, West China Hospital, Sichuan University, Chengdu, China

**Keywords:** cancer management, out-of-hospital, questionnaire survey, survival, Internet medical services

## Abstract

**Objective:** To explore the current situation of the out-of-hospital management of patients with cancer and evaluate the feasibility of Internet medical intervention outside the hospital in China.

**Methods:** The questionnaire was designed based on the investigators' clinical experience, literature data, and the Anderson Symptom Scale, and adopted a cross sectional survey method.

**Results:** Totally 1,171 qualified questionnaires were analyzed. The results showed that 92.7% of patients with cancer experienced varying degrees of out-of-hospital symptoms after treatment, and a third of them needed clinical intervention. Abnormal blood test results outside the hospital were basically consistent with the events that occurred during the hospitalization. One third of patients with cancer could not identify abnormal results. The primary approaches to solve these abnormalities were to seek guidance from the physician in charge or from nearby hospitals, but only 6.75% patients sought help online. More than half of the life or work of patients with cancer are still greatly affected under the current management model. 92% of respondents required medical help outside the hospital, and 65% ones were willing to pay for the out-of-hospital management.

**Conclusions:** Out-of-hospital management model needs to be improved. Most users are willing to accept Internet cancer management with fees. The survey has a positive effect on guiding future Internet cancer management practices in China to a certain extent.

## Background

Cancer management has been a heavy burden on the global healthcare system for a long time. The latest International Agency for Research on Cancer (IARC) GLOBOCAN cancer statistics reported that there were 19.29 million new cancer cases worldwide in 2020, of which 4.57 million new cancer cases were in China, accounting for 23.7% of the total, and 9.96 million deaths worldwide in 2020, of which 3 million deaths were in China, accounting for 30% of the total deaths (1). In addition, with the rapid development of anticancer drugs and medical technology, targeted therapy and immunotherapy have shown amazing curative effect, leading to the improvement of overall survival of patients with cancer. In 2020 alone, nearly 10 million cancer cases worldwide survived, and the cancer population continued to rise. Compared with other chronic diseases, the condition of patients with cancer tends to change greatly, resulting in more difficult disease management. Given that relying on the traditional cancer management remains an unmet clinical need, it is urgent to further explore new management models (2, 3).

The Internet is increasingly becoming an indispensable source of information for patients with cancer (4, 5). Satterlund et al. (6) reported that 49% of patients with breast cancer used the Internet for medical information up to 8 months after diagnosis, 40% of the patients used the Internet up to 16 months, and the Internet continued to provide medical information support for patients even until the end of the whole treatment. One study in Netherlands indicated that the proportion of patients with cancer using the disease related Internet increased dramatically from 60% in 2005 to 85% in 2017, and 47% of patients with cancer thought that the Internet was an important source of getting disease information (7). Additionally, Internet healthcare further expanded the content of services for patients including health education online, electronic health files, online consultation, online drug purchase, and other forms of health management services (8). Increasing online applications provided comprehensive health care services for patients with chronic diseases such as hypertension and diabetes, and they could also function in the field of postoperative follow up (4, 9–11). Furthermore, a number of prospective randomized controlled clinical trials abroad showed that patients with cancer accepted out-of-hospital follow up management with the help of Internet to report post-treatment reactions and symptoms, with significant benefits including emergency attendance reduction, improvement of life quality of patients with cancer, and longer overall survival (12–17). In this scenario, many countries are trying to build and improve out-of-hospital Internet management systems or applications suitable for most patients with cancer (18, 19).

Nowadays, the Internet is developing vigorously and has penetrated into the daily life of Chinese public. However, Chinese Internet medical services system of cancer management started late, and few reports of out-of-hospital management were issued, leading to a lack of management experience. Based on the above background, we performed this investigation to initially explore the current situation of out-of-hospital management of patients with cancer in Sichuan Province, China and analyze the cognition of Internet medical services in cancer management so as to provide some basis for better out-of-hospital management.

## Materials and Methods

### Study Population

A cross sectional study was conducted by convenience sampling method, and a total of 1,473 nonprimary inpatient patients with cancer meeting the criteria were selected from the departments of medical oncology of 13 tertiary hospitals in 10 localities in Sichuan Province from December 2020 to February 2021, respectively, for a web based questionnaire survey. These 13 hospitals were: Yibin Second People's Hospital, Nanchong Central Hospital, Xichang Yi Hospital, Panzhihua College Hospital, Chengdu Third People's Hospital, Chengdu Fifth People's Hospital, The First Hospital of Sichuan North Medical College, Neijiang Cancer Hospital, Suining Central Hospital, Zigong First People's Hospital, Zigong Fourth People's Hospital, Meishan Cancer Hospital and West China Hospital of Sichuan University. The main inclusion criteria of this study were: (i) 18 years old or older; (ii) with a clear histological or cytological diagnosis of malignancy and having had antitumor treatment (radiotherapy, chemotherapy, targeted therapy, or immunotherapy); (iii) with clear consciousness, normal comprehension, and good compliance to complete this questionnaire.

### Questionnaire Development

The questionnaire was designed through the researchers' clinical experience, expert evaluation as well as literature data, in which questions about patients' symptoms were designed with reference to the Anderson Symptom Scale. The final version of the questionnaire was revised repeatedly, and the electronic version was designed on the platform of “Questionnaire Star”.

### Data Collection (Questionnaire)

Specially trained investigators went to the wards of above mentioned major hospitals to assist the respondents to complete the questionnaire and answer patients' questions professionally.

### Quality Control

The subject principal officer conducted this investigation as a quality control officer, responsible for all questionnaires. Before carrying out, the subject research team performed a unified training of questionnaire collection personnel. After carrying out, the quality controller reviewed and checked the questionnaire [Questionnaire exclusion criteria were the following: (i) questionnaire filling time shorter than 120 s; (ii) questionnaire filling time beyond 8:00 am−10:00 pm].

### Statistical Methods

All questionnaires were downloaded from Questionnaire Star in excel, and the software Excel was used for initial data screening. SPSS 25.0 software was used for data analysis. The results were generally expressed in frequency numbers and percentages. Chi square test and Pearson correlation were performed according to the type of variables with *p* < 0.05 as a statistically significant difference.

### Ethical Consideration

The study protocol was reviewed and approved by the ethical review committees. All patients were fully informed, and they signed a written informed consent prior to the start of the study.

## Results

### Recovery and Reliability Test

From December 15, 2020 to February 24, 2021, a total of 1,171 (81.5%) valid questionnaires were analyzed after excluding 302 (20.5%) unqualified questionnaires. Questionnaire reliability and validity were analyzed by SPSS 25. The Cronbach's alpha coefficient was 0.747 (low reliability: α < 0.35; medium reliability: 0.35 < α < 0.70; high reliability: α >0.70) and the value of KMO was 0.805 (KMO > 0.9, very suitable for factor analysis; 0.8 < KMO < 0.9, suitable; >0.7, acceptable; = 0.6, with very poor effect; < 0.5, not suitable for factor analysis). The results indicated that the questionnaire was suitable for factor analysis and the overall validity was good.

### Basic Information

The median age of 1,171 patients was 57 years, with 6.2% in the 18–30 age group, 55.5% in the 31–60 age group, and 39.2% in the ≥61 age group. There were slightly more men than women. Those with junior high school education or below accounted for 58.41%. The proportion of those living in urban areas was 58.24%, and the types of diseases in this study were, in descending order, lung cancer, lymphoma or hematological disease, colorectal cancer, breast cancer, nasopharyngeal cancer, liver cancer, gastric cancer, and esophageal cancer. There were 96% of the survey respondents with medical insurance, the vast majority of whom had only basic medical insurance, among whom only 2.22% had commercial insurance, and 9.82% of patients had both insurance (Table 1).

**Table 1:** Demographic characteristics.

**Characteristics**		**Number** **of cases** **(*N* = 1,171)**	**Percentage** **(%)**
Tumor classification			
	Lung cancer	230	19.64%
	Lymphoma or other hematological cancers	153	13.07%
	Colorectal cancer	130	11.10%
	Breast cancer	109	9.31%
	Nasopharyngeal carcinoma	93	7.94%
	Liver cancer	89	7.60%
	Stomach cancer	72	6.15%
	Esophageal cancer	61	5.21%
	Cervical cancer	59	5.04%
	Prostate cancer	25	2.13%
	Other tumors	150	12.81%
Gender			
	Male	658	56.19%
	Female	513	43.81%
Age			
	≤ 30	62	5.30%
	31 60	651	55.50%
	≥61	458	39.20%
Education attainment			
	Junior high school or below	684	58.41%
	High school degree	272	23.23%
	Bachelor degree	200	17.08%
	Graduate degree or above	15	1.28%
Area of residence			
	City and town	682	58.24%
	Countryside	489	41.76%
Occupation			
	Farmer	395	33.73%
	Retirement	248	21.18%
	Worker	161	13.75%
	Other	367	31.34%
Insurance status			
	Basic medical insurance	987	84.29%
	Business insurance	26	2.22%
	Both	115	9.82%
	Neither	43	3.67%

### Patients' Out-of-Hospital Symptoms and Examination Abnormalities

**Figure 1:**
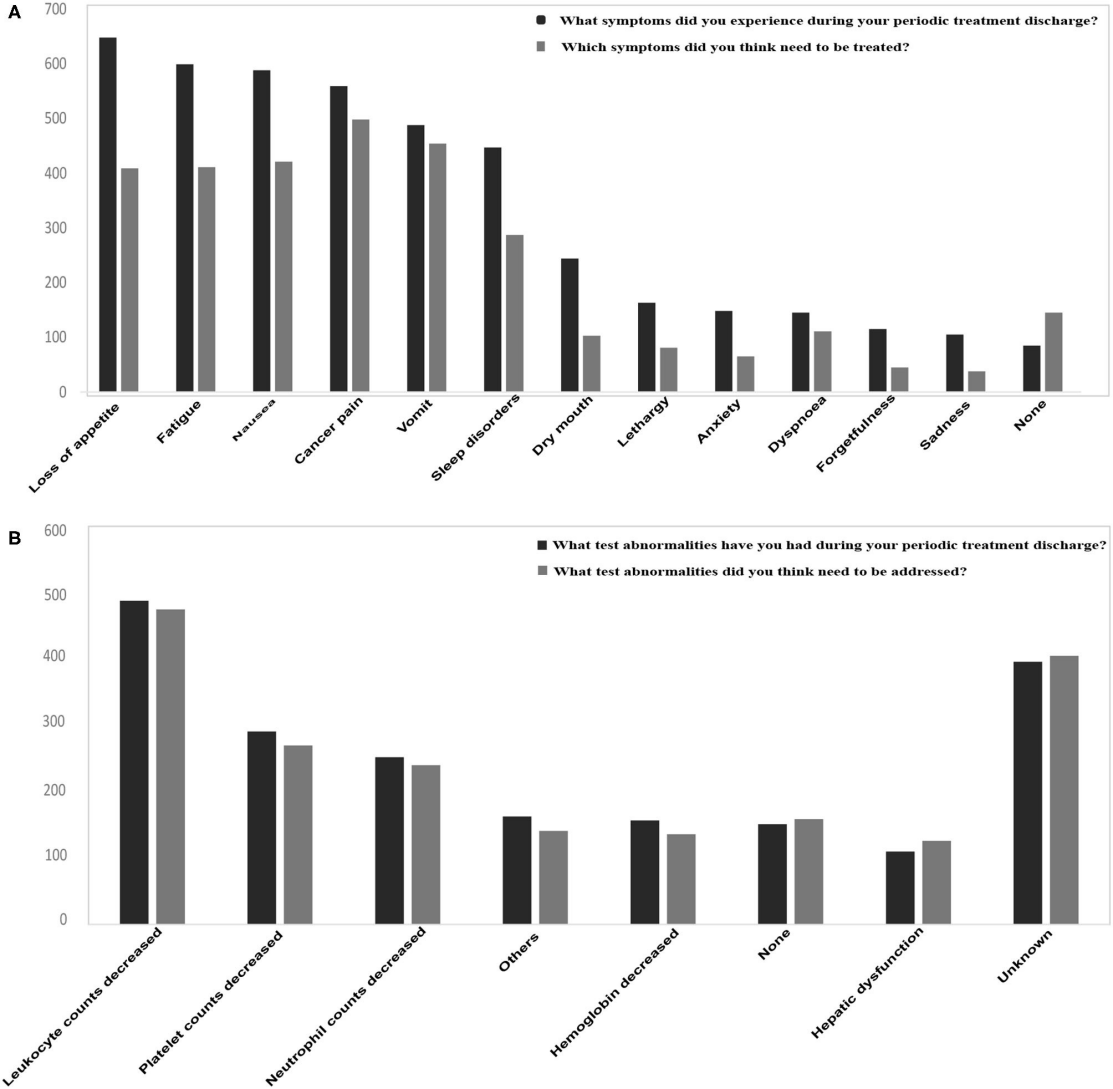
Patients, out-of-hospital symptoms **(A)** and examination **(B)** abnormalities.

According to the results, 92.74% of patients presented with various treatment or disease related out-of-hospital symptoms. The correlation analysis showed that those were mainly related to cancer type (*p* = 0.007), age (*p* < 0.001), and annual household income (*p* = 0.026), whereas gender, residence, health insurance status, occupation, and education attainment were not correlated (*p* > 0.05). Also, 87.62% of the patients thought that these symptoms needed to be treated. In terms of symptoms, the incidence exceeded 50%, including loss of appetite, fatigue and nausea, and 30–50% of the symptoms included pain, vomiting, and sleep disturbances. The proportion of patients with abnormal tests in routine out-of-hospital examinations was 53.29%, and 33.82% of patients were not sure of their test results or had no test results. Patients' attitudes toward different symptoms were different, for example, most patients thought that treatment was needed when they experienced pain and vomiting, whereas when symptoms such as dry mouth, fatigue, nausea, lethargy, and depression occurred, a considerable number of patients believed that treatment was not required (Figure 1A). However, when abnormal test indicators appeared, most patients thought that active intervention was required (Figure 1B). These abnormalities had an impact on the lives of 93.68% of patients, and 39.2% of them believed that they had a significant impact on their lives. At last, 92% of the respondents required out-of-hospital medical help, including symptom control (76.69%), interpretation of test reports (61.14%), dietary guidance (45.26%), sleep assistance (34.81%), and psychological counseling (25.36%).

### Internet Medical Use and Correlation Analysis

The survey showed that when these outpatients wanted medical help, the nearby hospitals and their treating doctors were still main choices of them, except for 11.87% of cases who handled the problem by themselves, and only 6.75% of cases who sought help from the Internet. The main forms were as follows: searching for medical content through search engines, asking other patients for solutions through social media, and seeking advice from nonsupervised physicians on the Internet. The majority (78.57%) of patients felt that seeking guidance from a competent physician was the most helpful way to solve their problem, whereas 14.09% of patients felt that going to the nearest doctor would be the most helpful, and only 1.88% approved of online medical help. The survey revealed that patients contacted their supervising physicians in a variety of ways, with the highest percentage making outpatient registration (64.22%) or going directly to the ward (58.5%). Correlation analysis showed that the use of Internet healthcare services was correlated with age (*p* = 0.018), education (*p* < 0.001), annual household income (*p* < 0.01), and occupation (*p* < 0.001), and not with gender (*p* = 0.302), residence (*p* = 0.059), or health insurance status (*p* = 0.254) (Table 2).

**Table 2:** Correlation analysis of the use of internet healthcare.

**Characteristics**		**Internet** **access to** **healthcare**	**Non** **Internet** **access**	***P***
Age^a^		–	–	0.018
Education attainment^a^		–	–	<0.001
Annual household income^a^		–	–	0.005
Gender^b^				0.302
	Male	40	618	
	Female	39	474	
Residence^b^				0.059
	Cities and towns	53	628	
	Countryside	25	464	
Medical insurance status^b^				0.254
	Purchase of basic medical insurance	62	925	
	Purchase of commercial insurance	3	23	
	Both	12	103	
	Neither	2	41	
Occupation^b^				<0.001
	Farmer	11	384	
	Retirement	19	229	
	Worker	14	147	
	Other	35	332	

a *Pearson correlation was employed for correlation analysis between continuous or ordered categorical variables and categorical variables*;

b *Chi square test was used for correlation analysis between dichotomous or unordered categorical variables and categorical variables*.

### Analysis of Cancer Patients' Awareness of Internet Healthcare and Correlations

**Figure 2:**
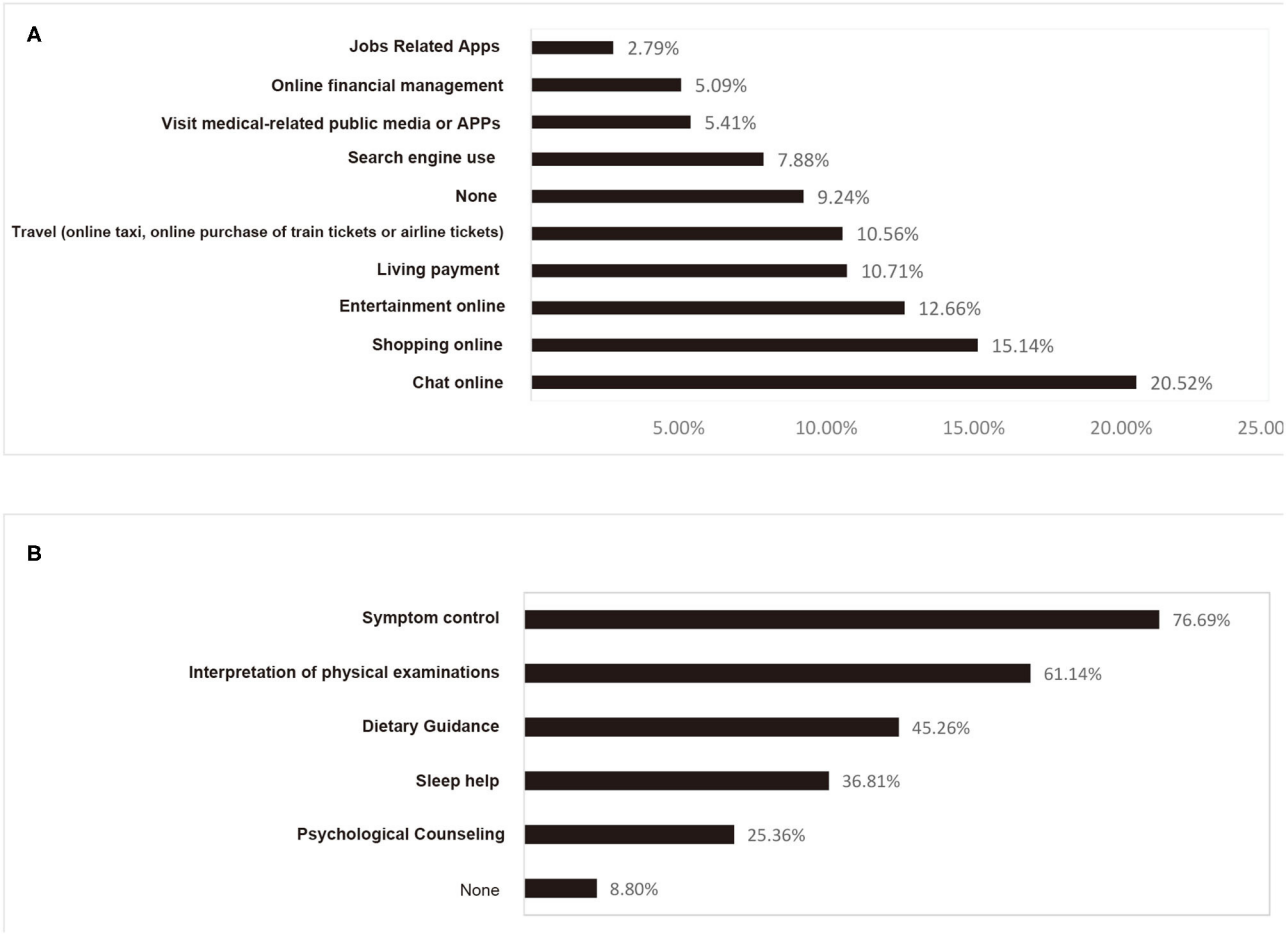
Proportion of the public using the Internet in various aspects **(A)** and the medical needs that patients expect outside the hospital **(B)**.

As shown in Figure 2A, the public daily used the Internet mainly for shopping (15.14%), entertainment (12.66%), and chatting (20.52%), while only 5.41% used it in relation to e-healthcare, with correlations including age (*p*< *0*.001), education (*p* < 0.001), and annual household income (*p*< *0*.001). The current medicine related services on the Internet were mainly categorized into three types: social communication groups online established by patients spontaneously, the use of search engines, and Internet medical consultation services. The survey revealed that patients considered the main advantages of communication groups as information sharing (27.87%), mutual comfort, and encouragement (29.07%), as well as the disadvantages mainly included cumbersome information (36.93%), low credibility of content (26.27%), and too much negative information (25.73%). The main advantages of self searching were convenient (30%), free of charge (25.91%), and fast (25.16%), while the disadvantages included worse credibility (45.7%), too much professionalism (29.46%), and too many advertisements (27.31%). The main advantages of online consultation were mainly convenience (42.7%) and high credibility (24.77%), and the disadvantages mainly included no timely feedback (37.57%), improper judgement on their condition from the consultation by non-supervisory doctors (51.49%), and highly expensive fees (16.57%) (Table 3). In addition, the study showed that 65% of the respondents were willing to pay the fees for Internet medical intervention for out-of-hospital management, requiring more out-of-hospital medical help (Figure 2B). Further correlation analysis showed that the correlation of willingness to pay the fees for Internet healthcare services included tumor category (*p* = 0.023), age (*p* < 0.001), area of residence (*p* < 0.001), education (*p* < 0.001), health insurance status (*p* < 0.001), and occupation (*p* = 0.015), but not gender (*p* = 0.119) and annual household income (*p* = 0.084) (Table 4).

**Table 3:** Evaluation of domestic internet medical applications by oncology patients.

**Projects**		**Number of cases**	**Percentage**	**Projects**		**Number of cases**	**Percentage**
Advantages of communication group online	Information sharing	209	27.87%	Disadvantages of outpatient group	Information complexity	277	36.93%
	Mutual encouragement	218	29.07%		Lack of credibility	197	26.27%
	Authentic	87	11.60%		Too much negative information	193	25.73%
	Other	229	30.53%		Other	434	57.87%
Advantages of self searching	Convenient	279	30%	Disadvantages of self search	Worse Credibility	425	45.70%
	Free	241	25.91%		Can't understand professional information	274	29.46%
	Fast	234	25.16%		Too many advertisements	254	27.31%
	Other	270	29.03%		Other	438	47.10%
Advantages of online consultation	Professional and credible	290	24.77%	Disadvantages of online consultation	Expensive charges	194	16.57%
	Convenient and fast	500	42.70%		Non supervising physicians	603	51.49%
	Other	320	27.33%		Lack of timely feedback	440	37.57%
					Other	459	39.20%

**Table 4:** Correlation analysis of willingness to pay for medical treatment on the Internet.

**Characteristics**		**Reluctance**	**Willingness**	***P***
Age^a^		–	–	<0.001
Education attainment^a^		–	–	<0.001
Annual household income^a^		–	–	0.084
Tumor category^b^		–	–	0.023
	Lung cancer	79	151	
	Lymphoma or other blood disorders	55	98	
	Colorectal cancer	39	91	
	Breast cancer	29	80	
	Nasopharyngeal carcinoma	40	53	
	Other	168	288	
Gender^b^				0.119
	Male	243	415	
	Female	167	346	
Residence^b^				<0.001
	Cities and towns	200	482	
	Countryside	210	279	
Medical insurance status^b^				<0.001
	Purchase of basic medical insurance	361	626	
	Purchase of commercial insurance	9	17	
	Both	14	101	
	Neither	26	17	
Occupation^b^				
	Farmers	157	238	0.015
	Retirement	91	157	
	Workers	49	112	
	Other	113	254	

a *Pearson correlation was employed for correlation analysis between continuous or ordered categorical variables and categorical variables*;

b *Chi square test was used for correlation analysis between dichotomous or unordered categorical variables and categorical variables*.

## Discussion

This study investigated the out-of-hospital conditions of patients with cancer in 13 large scale hospitals in Sichuan Province and found that up to 92.7% of patients with cancer still had different degrees of out-of-hospital symptoms after the treatment, and 53.3% of patients had abnormal test indicators. Of note, up to one third of the patients did not understand their test results or they did not know how to monitor it. These anomalies affected the life of most patients with cancer, and also 92% of respondents expected to receive appropriate out-of-hospital medical help.

Patients with cancer have significant differences in cognition of disease related symptoms and abnormal indicators outside the hospital. Most patients believe that vomiting, pain, virtually all of test abnormalities and so on need to be intervened, while loss of appetite, mood abnormalities, and insomnia symptoms do not need too much attention, which is quite different from the understanding of oncologists. Oncologists suggest that many of the test abnormalities are not necessarily required for special treatment, while symptoms such as loss of appetite and mood abnormalities are considered to require adequate attention. Therefore, if the nonhospital patients are not well managed, it may lead to unnecessary or inadequate intervention, ultimately affecting the treatment effectiveness and the quality of life of the patient. Miller et al. (20) believed that good cancer management can improve the condition of the patients including psychological, behavioral, and physical aspects. Moreover, Basch et al. (17) designed the out-of-hospital management app (Application) for patients with cancer and conducted prospective randomized controlled clinical trials, and the results revealed that based on patients reporting symptoms *via* the Internet, cancer out-of-hospital follow up management not only improved the patient survival quality, but also increased the overall survival. An observational study for patients with breast cancer in China also observed that Internet users satisfied with Internet information had a longer disease-free life (21). Interestingly, we also found that patients tend to report that nausea makes them more miserable, but vomiting is more likely to require intervention. Also, there are some differences between drowsy and fatigue. Drowsy tends to reflect their mental state, while fatigue tends to reflect their physical energy. The causes of the two symptoms are different: drowsy may be caused by an adverse reaction to a chemical agent and fatigue may be caused by hypoglycemia, hypokalemia, leukopenia, etc. People traditionally think that it is common to associate nausea with vomiting. However, in fact, there is definitely a difference between these symptoms, which is consistent with clinical observation from oncologists. Taken together, the Internet plays a non-negligible role in cancer management.

Our work also observed that about 90% of patients with cancer in Sichuan used the Internet on a routine basis, and only 5.41% of them daily used the Internet for medical help, and they are mainly young and highly educated. Other studies have also reported a clear correlation between online search for tumor information and their age and educational attainment in patients with breast cancer (11, 21). The main reasons why patients remain skeptical about the use of Internet medical information include: unreliable content, difficulty in distinguishing valid information by themselves, and poor judgment of the condition during consultation with unfamiliar physicians. Other studies have reported that patients with breast cancer are able to understand breast cancer related Internet information less, and many websites are commercial in nature, questioning the credibility of their information (22). As a result, most patients tended to seek guidance from a familiar physician. Of note, in the communication patterns with familiar doctors, we found that in addition to the traditional face to face medical model, there has been a certain proportion of physicians using the Internet to manage their patients. As a complement to the traditional medical model, this approach is more easily accepted by the young, highly educated, and high family income groups. The patient occupation also has a certain correlation with Internet communication and management. A survey of the attitudes of patients with cancer toward online healthcare showed that patients with graduate degrees and higher incomes are more eager to access cancer information online, especially for high quality information (23).

For Internet out-of-hospital management with fees, 65% of patients with cancer accept it, and and there is a clear correlation between tumor type, age, region of residence, education attainment, medical insurance status and patient occupation. Moreover, our analysis indicated that the annual household income of patients is not related to the willingness to pay for Internet medical treatment. A significant proportion of patients with cancer are unable to visit hospitals regularly for follow up and symptom management due to various causes such as geographical distance, economic difficulties, lack of medical resources, and insufficient social support. However, for families with affluent income, it is easier to adhere to the traditional face-to-face medical model. In terms of needs, the main needs included symptom control, interpretation of examination and test reports, dietary guidance, sleep regulation, and psychological counseling, which also showed that patients with cancer wanted to receive more attention outside the hospital. If the authenticity and convenience of cancer information from Internet healthcare can be guaranteed through payment, patients are willing to accept it. This is a guiding meaning for future cancer Internet medical application practice.

As this work is confined to Sichuan Province, the population may not be representative of the entire Chinese population. In addition, this study is a questionnaire study with a small sample size, thereby a multicenter study with a larger cancer population would increase credibility of these results.

In summary, the incidence of cancer related out-of-hospital symptoms is high, and the out-of-hospital management needs to be improved. In view of a large demand for medical assistance, a high Internet usage rate, a certain understanding of Internet medical treatment, and the willingness to accept payment for Internet medical care, the application of Internet medical care in the cancer out-of-hospital management has a certain feasibility.

## Data Availability Statement

The raw data supporting the conclusions of this article will be made available by the authors, without undue reservation.

## Ethics Statement

The studies involving human participants were reviewed and approved by Ethics Committee of Clinical Trials and Biomedical, West China Hospital, Sichuan University. The patients/participants provided their written informed consent to participate in this study.

## Author Contributions

SD and XL were responsible for the methodology, formal analysis, and preparation of the initial manuscript draft and the final manuscript. JB, CD, JM, and XC were responsible for data collection and contributed to the analysis interpretation. MJ was responsible for the conceptualization, methodology, and review of the final manuscript.

## Conflict of Interest

The authors declare that the research was conducted in the absence of any commercial or financial relationships that could be construed as a potential conflict of interest.

## Publisher's Note

All claims expressed in this article are solely those of the authors and do not necessarily represent those of their affiliated organizations, or those of the publisher, the editors and the reviewers. Any product that may be evaluated in this article, or claim that may be made by its manufacturer, is not guaranteed or endorsed by the publisher.
